# Supplementary material for: Quantifying H5N1 outbreak potential and control effectiveness in high-risk agricultural populations
Source: PLOS Glob Public Health. 2025 Dec 29;5(12):e0005463. doi: 10.1371/journal.pgph.0005463 (PMC12747336; doi:10.1371/journal.pgph.0005463)
Supplement: S1 File — (DOCX) [file pgph.0005463.s001.docx]

**S1 File. Pre-processing of CoMix data**

We used CoMix data to compare contact patterns with those from the Avian Contact Study, which is publicly available [here](https://zenodo.org/records/11154066). Specifically, we used the “participant_common” and “contact_common” datasets to obtain the number of contacts reported by each participant. The “participant_common” dataset includes information such as participant id, age group, and gender, while the “contact_common” dataset is in wide format and contains participant id, contact id, and details about each contact.

We extracted UK data, and included only participants aged above 18, as the Avian Contact Study recruited individuals over 18 living in the UK. We calculated the total number of contacts for each participant by counting the appearance of each unique participant id in the “contact_common” data. Note that participants with zero contacts do not appear in this dataset. We identified these participants from “participant_common” and assigned them a contact count of zero. Age group of the participants was also obtained from the “participant_common” dataset.

For consistency with the Avian Contact Study, we applied a maximum cut-off of 20 contacts per participant and used those contact counts in our analysis.
