# Supplementary material for: Quantifying H5N1 outbreak potential and control effectiveness in high-risk agricultural populations
Source: PLOS Glob Public Health. 2025 Dec 29;5(12):e0005463. doi: 10.1371/journal.pgph.0005463 (PMC12747336; doi:10.1371/journal.pgph.0005463)
Supplement: S2 File — (DOCX) [file pgph.0005463.s002.docx]

**S2 File. Comparing contact data from the Avian Contact Study to CoMix**

We applied various methods to compare the contact patterns between the Avian Contact Study and CoMix. We provided a visual comparison of the probability distributions of the contact patterns in S1 Fig. The CoMix dataset showed higher probabilities for 0 to 3 contacts, while the Avian Contact Study showed higher probabilities for 4 to 12 contacts.

We also used statistical tests to compare the patterns, such as a chi-square test on grouped contact counts. We grouped the number of contacts into the following categories: 0-1, 2, 3, 4, 5, 6, 7-9, 10-20. This ensured that the expected frequency for each category is at least 5, which is one of the assumptions of the chi-square test. We also calculated the Total Variation Distance (TVD), defined as $\frac{1}{2}\sum|P\left( x \right)-Q\left( x \right)|$, where $P\left( x \right)$and $Q\left( x \right)$ are the probability distributions of the Avian Contact Study and CoMix, respectively. We then used a permutation test to assess the statistical significance of the estimated TVD by randomly shuffling the survey labels 10000 times. This produced a distribution of TVD values that would be expected if there was no difference between the groups. The p-value was then calculated by determining the proportion of TVD values in the permutation distribution that are greater than or equal to the observed value. We estimated the TVD to be 0.31, and the p-values from both the chi-square test and the permutation test less than 0.0001.

Overall, these findings suggest a statistically significant moderate difference in contact distributions between the two datasets. These differences may be due to differences in the settings of both surveys, such as the study periods and the target population.
