# Supplementary material for: Quantifying H5N1 outbreak potential and control effectiveness in high-risk agricultural populations
Source: PLOS Glob Public Health. 2025 Dec 29;5(12):e0005463. doi: 10.1371/journal.pgph.0005463 (PMC12747336; doi:10.1371/journal.pgph.0005463)
Supplement: S1 Fig — (DOCX) [file pgph.0005463.s003.docx]

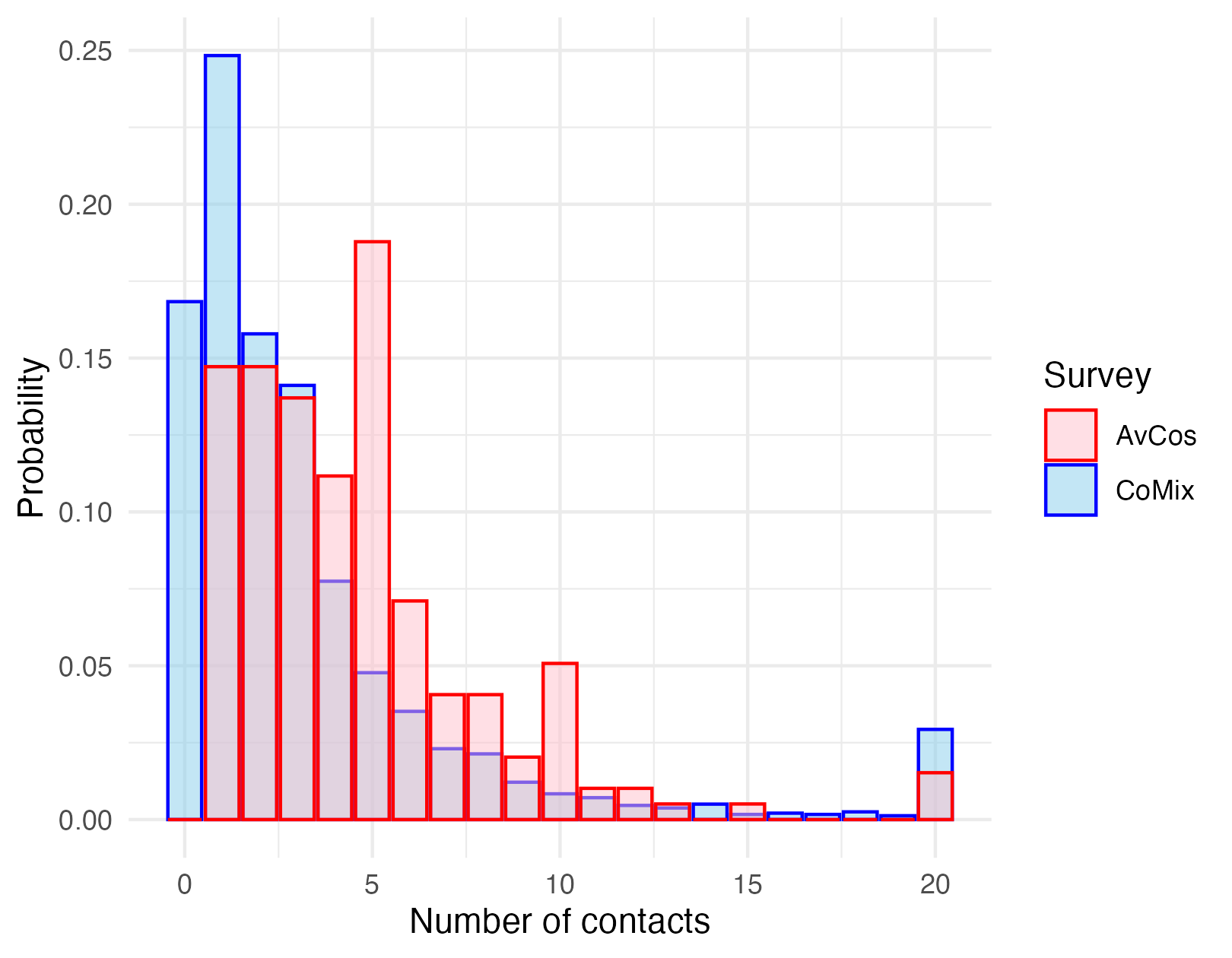
**S1 Fig**

**S1 Fig.** **Comparison of the empirical probability distributions of the number of contacts reported in the Avian Contact Study (AvCos) and CoMix.**
