# Supplementary material for: Quantifying H5N1 outbreak potential and control effectiveness in high-risk agricultural populations
Source: PLOS Glob Public Health. 2025 Dec 29;5(12):e0005463. doi: 10.1371/journal.pgph.0005463 (PMC12747336; doi:10.1371/journal.pgph.0005463)
Supplement: S2 Fig — (DOCX) [file pgph.0005463.s004.docx]

**S2 Fig**

**
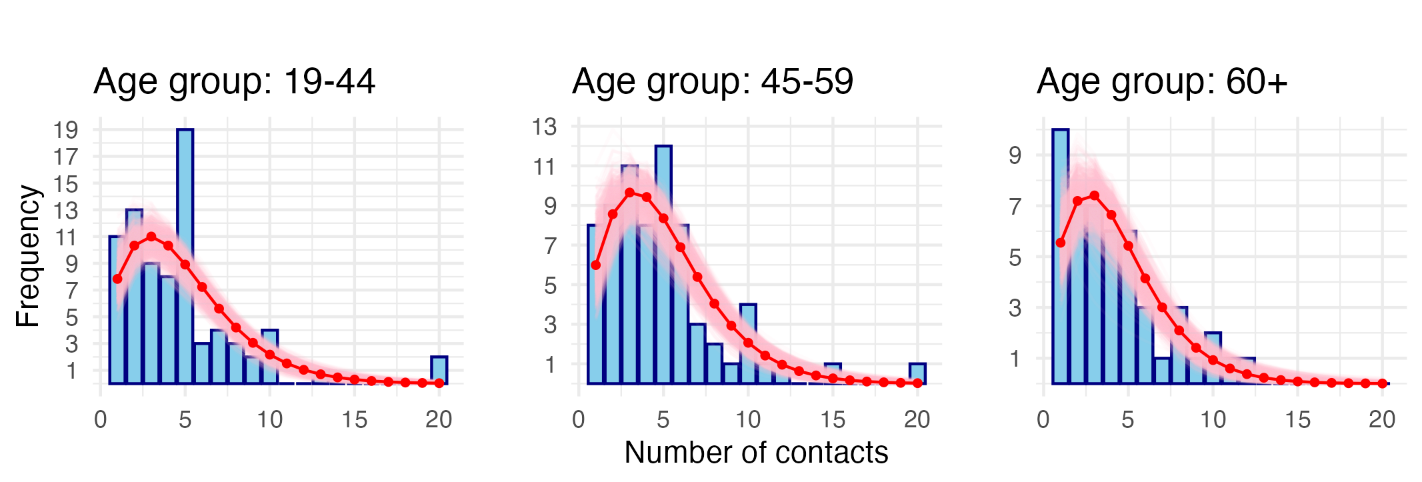
**

**S2 Fig.** **Distribution of age-stratified number of contacts from the Avian Contact Study**. The red line indicates the fitted negative binomial distribution, and the pink region represents the 95% confidence interval.
