# Supplementary material for: Quantifying H5N1 outbreak potential and control effectiveness in high-risk agricultural populations
Source: PLOS Glob Public Health. 2025 Dec 29;5(12):e0005463. doi: 10.1371/journal.pgph.0005463 (PMC12747336; doi:10.1371/journal.pgph.0005463)
Supplement: S3 Fig — (DOCX) [file pgph.0005463.s005.docx]

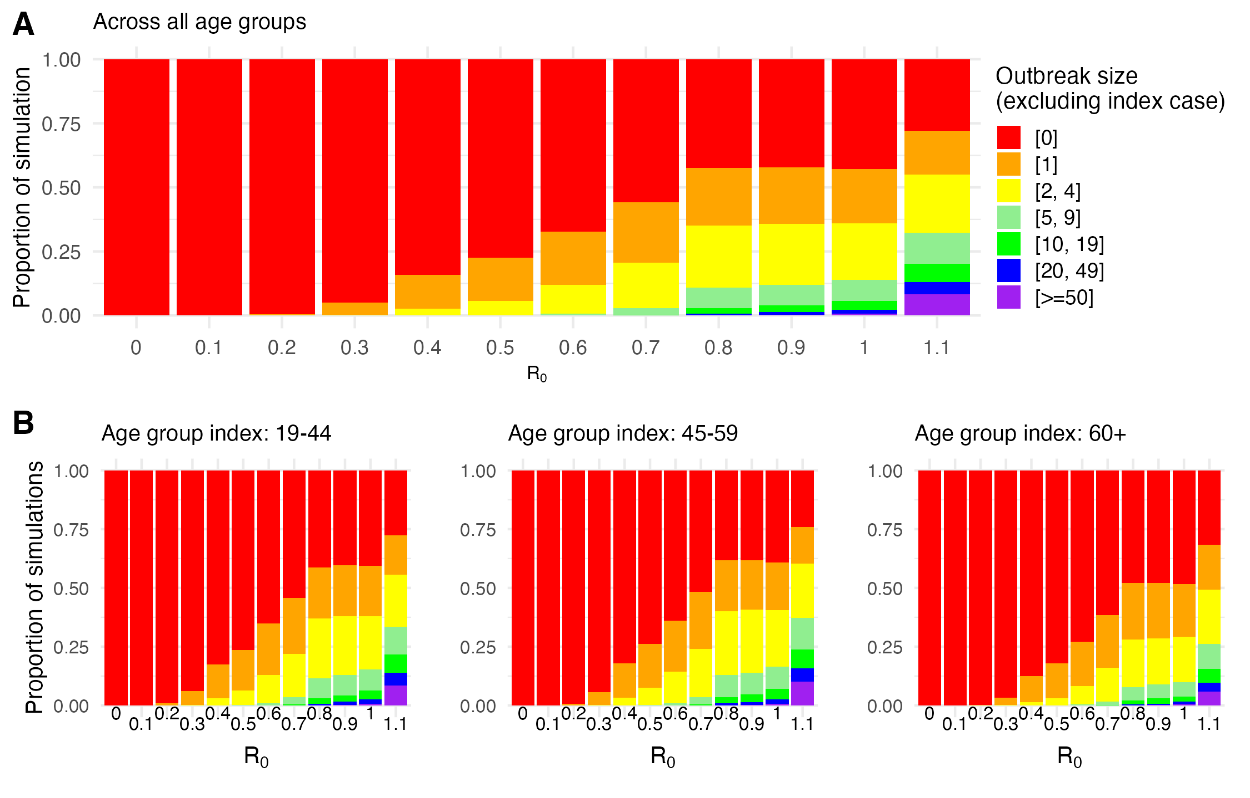
**S3 Fig**

**S3 Fig.** **Effect of age and basic reproduction number on final outbreak size.** **(A)** Outbreak size across all age group. **(B)** Age-specific outbreak size. Each coloured bar represents a different outbreak size (excluding the index case), and the height of each bar indicates the proportion of simulations resulting in the corresponding outbreak size. We assumed 40% of cases were asymptomatic.
