# Supplementary material for: Quantifying H5N1 outbreak potential and control effectiveness in high-risk agricultural populations
Source: PLOS Glob Public Health. 2025 Dec 29;5(12):e0005463. doi: 10.1371/journal.pgph.0005463 (PMC12747336; doi:10.1371/journal.pgph.0005463)
Supplement: S4 Fig — (DOCX) [file pgph.0005463.s006.docx]

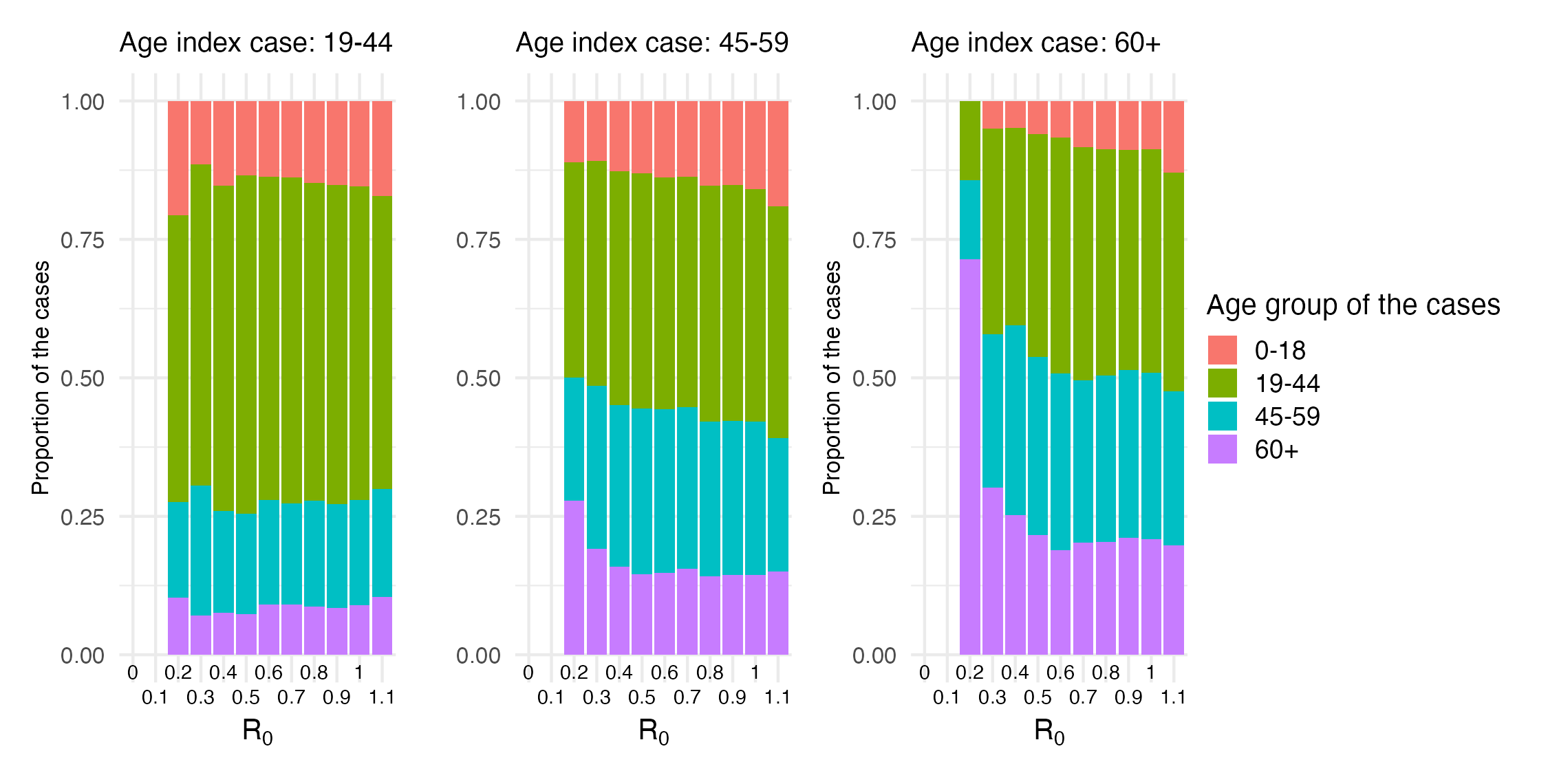
**S4 Fig**

**S4 Fig.** **Impact of age of the index case on the age distribution of cases in a cluster.** Each coloured bar represents a different age group, and the height indicates the average proportion of cases resulting in that age group across 9000 simulations. No bars are shown for R_0_ values of 0 and 0.1, as no secondary cases occurred at those values. We assumed 40% of cases were asymptomatic.
