# Supplementary material for: Quantifying H5N1 outbreak potential and control effectiveness in high-risk agricultural populations
Source: PLOS Glob Public Health. 2025 Dec 29;5(12):e0005463. doi: 10.1371/journal.pgph.0005463 (PMC12747336; doi:10.1371/journal.pgph.0005463)
Supplement: Attachment — Submitted filename: rebuttal_letter.docx [file pgph.0005463.s008.docx]

Quantifying H5N1 outbreak potential and control effectiveness in high-risk agricultural populations

We would like to thank the reviewers and editors for the positive and considered reviews. We have revised the manuscript following the comments and provide a point-by-point response below in blue.

Reviewers' comments:

The study addresses the ongoing H5N1 panzootic, a topic of major global health concern. By focusing on

zoonotic spillover and potential human-to-human transmission, it connects well to pressing pandemic

preparedness questions. Here are my suggestions:

The study acknowledges asymptomatic cases but doesn’t deeply explore realistic ranges of asymptomatic

infection in H5N1. Since asymptomatic carriage in humans is poorly understood, exploring a wider range of

assumptions (from very low to moderate prevalence) would add robustness. Maybe authors can discuss this

point

Thank you for all the questions and suggestions. We agree that asymptomatic infection with H5N1 in humans is poorly understood, as noted in the introduction at lines 61-63. To address this uncertainty, we explored values ranging from 0% to 100% in 20% intervals to investigate the impact of asymptomatic infections on the effectiveness of control measures. We have clarified this in the methods under “Asymptomatic infections” at lines 205-213 and presented the results in Figure 4B. We have deepened our discussion at lines 335-337.

“Our model provides insights into the potential outbreak size of avian influenza among humans under different levels of transmission and a full range of proportion of asymptomatic infections to account for uncertainties around these values.”

While the UK setting is clear, the contact structures and public health response capacity differ in low- and

middle-income countries where zoonotic spillover risk is high. Discussion of transferability would broaden

the relevance.

We have added this in the “Strengths and limitations” subsection of the discussion at lines 392-398, as follows:

“Contact patterns may differ in low- and middle-income countries, where intergenerational living and higher contact rates among older adults are more common. Furthermore, a study by Eze et al. comparing epidemic preparedness and response capacity across 186 countries identified countries with lower response capacity, most of which are low- and middle-income countries. Hence, our findings may not be generalised to other countries with different contact structures or limited diagnostic and contact tracing resources.”

The agricultural contact data are valuable, but heterogeneity within and across communities (e.g., multi-

generational households, seasonal work, market interactions) could have been discussed more fully. This

heterogeneity may affect outbreak potential.

Thank you for raising this point. We agree that understanding how contact patterns vary by covariates such as household structure, size, seasonal working patterns, and market interactions is valuable. Although we have a relatively small sample, focused on poultry farmers, comparison with other social contact data and qualitative knowledge of farming communities suggests that the data reveal robust patterns. Our estimates are consistent with CoMix, and further, we find strong intergenerational household mixing, reflecting the multi-generational farming enterprises. The differences we identify warrants further exploration of farming contact patterns. We have included this point as an area for future work in the discussion at lines 406-421, as follows:

“Our sample was relatively homogeneous in the occupational group, with most respondents being poultry farmers. In addition to farmers, who make up 60% of the agricultural workforce in the UK, 12% are casual workers involved in seasonal work and often exhibiting different living arrangements. Despite their high exposure risk and potential influence on outbreak dynamics, it can be challenging to engage with this group due to language and trust barriers. Hence, future research is required to further characterise contact patterns within agricultural demographics and develop effective strategies to engage with groups that are underrepresented in contact surveys.”

Only contact tracing and self-isolation are modeled. In reality, outbreak management could include infection

control, health care facility and capacity, movement restrictions, or culling of infected animals. Considering

at least one additional intervention would make the study more comprehensive.

Our motivation to explore only contact tracing and self-isolation was guided by current public health guidance implemented by the UK public health teams (<https://www.gov.uk/government/publications/avian-influenza-managing-human-exposures-to-incidents-in-birds-or-animals/investigation-and-initial-clinical-management-of-possible-human-cases-of-avian-influenza-with-potential-to-cause-severe-human-disease>). Similar control measures are also recommended by the US CDC and the European Centre for Disease Prevention and Control. While we agree that movement restrictions and culling of infected animals are effective strategies to contain outbreaks in animal reservoirs, our model focuses explicitly on human-to-human transmission following a single spillover event and is therefore unaffected by transmission in the animal reservoir.

Other infection control measures, such as hand hygiene or use of personal protective equipment were reported by over 90% of respondents, so would be unlikely to make a substantial difference to our results without further data quantifying risk reductions. These measures primarily reduce the risk of spillover rather than human-to-human transmission. Although we did not model asymptomatic testing, we showed the limitations of current interventions for asymptomatic infections and highlighted the importance of asymptomatic testing as an additional control strategy in the discussion at lines 340-356.

The study convincingly demonstrates that early interventions like contact tracing and self-isolation can

substantially reduce outbreak size when R**₀** is low and symptomatic detection is reliable. However, if R**₀**

increases or asymptomatic transmission is significant, these interventions may not suffice. Authors can

discuss this point

We have discussed the reduced effectiveness of contact tracing and self-isolation with an increasing proportion of asymptomatic infections and higher $R_{0}$ values in the discussion at lines 342-345.

“Contact tracing and self-isolation reduced outbreak size, but their potential effectiveness in preventing cases decreased as the proportion of asymptomatic infections increased, since they rely on symptomatic cases. Additionally, larger values of $R_{0}$ would increase outbreak size and the number of contacts, which may slow down contact tracing efforts and reduce the effectiveness of current interventions.”

For policymakers, this suggests that contact tracing and self-isolation are valuable but fragile tools—effective

only under certain epidemiological conditions. Maybe authors can discuss they should be embedded in a

layered response strategy including rapid diagnostics, surveillance, and (eventually) vaccines or antivirals.

We have expanded on this point in discussion and the “Translating findings into action” subsection at lines 349-356, as follows:

“Limited understanding of the proportion of asymptomatic H5N1 infections and the factors influencing symptomatology, combined with their potential to reduce the effectiveness of contact tracing and self-isolation, highlights that these measures, while valuable, are vulnerable. Embedding them within a layered response strategy that includes asymptomatic testing programmes, which have previously shown to be successful at detecting cases, could enhance current interventions.”

Editor comments:

Given that there is little to no evidence of human-to-human transmission for avian influenza (H5N1), is self-

isolation recommended as a control measure for human cases? Additionally, is self-isolation applicable in the

context of seasonal influenza as well?

Thank you for your questions. Yes, contacts of confirmed human cases of avian influenza who develop symptoms are assessed as a possible cases and are advised to self-isolate while arrangements are made for clinical assessment and investigation, according to UKHSA guidance (<https://www.gov.uk/government/publications/avian-influenza-managing-human-exposures-to-incidents-in-birds-or-animals/investigation-and-initial-clinical-management-of-possible-human-cases-of-avian-influenza-with-potential-to-cause-severe-human-disease>) Contact tracing and self-isolation are also advised by the US CDC and ECDC.

There is no such explicit guidance for seasonal influenza.

Introduction section: Line number 66: However, cases without zoonotic exposure and limited human-to-

human transmission have been documented. Specify the virus name. Seasonal or avian influenza.

We have specified the virus name as H5N1 at line 69.

In method: you mentioned "contact with birds". It is better to mention the name bird or poultry or chicken

or turkey. The meaning of bird is different than poultry.

The questionnaire was open to individuals who have contact with domestic and/or wild birds. As it was launched at the Pig and Poultry Fair, most of the respondents were poultry farmers who had contact with poultry, mostly chickens. We have clarified this in the “Data description” section at lines 110-111, as follows:

“Most respondents were poultry farmers, reporting contact with poultry, mainly chickens (82%).”

Does the model possess adequate capability to address avian influenza, considering the virus exhibits

limited human-to-human transmissibility?

We used a branching process model as the most appropriate modelling framework for capturing infections with limited human-to-human transmission. A branching process model is able to capture a full range of scenarios from no human-to-human transmission to sustained human transmission, although alternative modelling framework might be more appropriate in situations with widespread community transmission. Our intention was to focus on an early outbreak phase with limited human-to-human transmission when the susceptible population is large. This approach allows us to simulate stochastic extinction and outbreak variability, which is well-suited to emerging infections with limited transmissibility, such as H5N1. The model therefore reflects the current epidemiological understanding of H5N1, with limited human-to-human transmission and the potential for asymptomatic infections. We explored a range of basic reproduction number values consistent with other pandemic influenza viruses for different transmission scenarios.

We have expanded on our reasoning in the “Model framework” at lines 163-164, as follows:

“We used a branching process model to simulate early-stage outbreaks, as it can capture stochastic extinction and outbreak variability for infections with limited human-to-human transmission.”

We thank the reviewers and editors for their constructive feedback, which has helped improve the manuscript. We hope our responses and revisions address the points raised, and we would be happy to provide any additional clarification if required.
