# Supplementary material for: Quantifying H5N1 outbreak potential and control effectiveness in high-risk agricultural populations
Source: PLOS Glob Public Health. 2025 Dec 29;5(12):e0005463. doi: 10.1371/journal.pgph.0005463 (PMC12747336; doi:10.1371/journal.pgph.0005463)
Supplement: Peer review history

# Peer review: Decision Letter 0

**DOI:** 10.1371/journal.pgph.0005463.r001

**Authors:** Sukanta Chowdury

13 Oct 2025

PGPH-D-25-02162

***Quantifying H5N1 outbreak potential and control effectiveness in high-risk agricultural populations***

PLOS Global Public Health

Dear Dr. Izel Avkan,

Thank you for submitting your manuscript to PLOS Global Public Health. After careful consideration, we feel that it has merit but does not fully meet PLOS Global Public Health’s publication criteria as it currently stands. Therefore, we invite you to submit a revised version of the manuscript that addresses the points raised during the review process.

Please submit your revised manuscript by 30th October 2025. If you will need more time than this to complete your revisions, please reply to this message or contact the journal office at globalpubhealth@plos.org. When you're ready to submit your revision, log on to https://www.editorialmanager.com/pgph/ and select the 'Submissions Needing Revision' folder to locate your manuscript file.

Please include the following items when submitting your revised manuscript:

- A rebuttal letter that responds to each point raised by the editor and reviewer(s). You should upload this letter as a separate file labeled 'Response to Reviewers'.
- A marked-up copy of your manuscript that highlights changes made to the original version. You should upload this as a separate file labeled 'Revised Manuscript with Track Changes'.
- An unmarked version of your revised paper without tracked changes. You should upload this as a separate file labeled 'Manuscript'.

Guidelines for resubmitting your figure files are available below the reviewer comments at the end of this letter.

We look forward to receiving your revised manuscript.

Kind regards,

Sukanta Chowdury, Ph.D

Academic Editor

PLOS Global Public Health

Journal Requirements:

1. Please note that PLOS Global Public Health has specific guidelines on code sharing for submissions in which author-generated code underpins the findings in the manuscript. In these cases, all author-generated code must be made available without restrictions upon publication of the work. Please review our guidelines at https://journals.plos.org/globalpublichealth/s/materials-and-software-sharing#loc-sharing-code and ensure that your code is shared in a way that follows best practice and facilitates reproducibility and reuse.

Reviewers' comments:

The study addresses the ongoing H5N1 panzootic, a topic of major global health concern. By focusing on zoonotic spillover and potential human-to-human transmission, it connects well to pressing pandemic preparedness questions. Here are my suggestions

The study acknowledges asymptomatic cases but doesn’t deeply explore realistic ranges of asymptomatic infection in H5N1. Since asymptomatic carriage in humans is poorly understood, exploring a wider range of assumptions (from very low to moderate prevalence) would add robustness. Maybe authors can discuss this point

While the UK setting is clear, the contact structures and public health response capacity differ in low- and middle-income countries where zoonotic spillover risk is high. Discussion of transferability would broaden the relevance.

The agricultural contact data are valuable, but heterogeneity within and across communities (e.g., multi-generational households, seasonal work, market interactions) could have been discussed more fully. This heterogeneity may affect outbreak potential.

Only contact tracing and self-isolation are modeled. In reality, outbreak management could include infection control, health care facility and capacity, movement restrictions, or culling of infected animals. Considering at least one additional intervention would make the study more comprehensive.

The study convincingly demonstrates that early interventions like contact tracing and self-isolation can substantially reduce outbreak size when R₀ is low and symptomatic detection is reliable. However, if R₀ increases or asymptomatic transmission is significant, these interventions may not suffice. Authors can discuss this point

For policymakers, this suggests that contact tracing and self-isolation are valuable but fragile tools—effective only under certain epidemiological conditions. Maybe authors can discuss they should be embedded in a layered response strategy including rapid diagnostics, surveillance, and (eventually) vaccines or antivirals.

Editor comments:

- Given that there is little to no evidence of human-to-human transmission for avian influenza (H5N1), is self-isolation recommended as a control measure for human cases? Additionally, is self-isolation applicable in the context of seasonal influenza as well?

- Introduction section: Line number 66: However, cases without zoonotic exposure and limited human-to-human transmission have been documented. Specify the virus name. Seasonal or avian influenza.

- In method: you mentioned "contact with birds". It is better to mention the name bird or poultry or chicken or turkey. The meaning of bird is different than poultry.

- Does the model possess adequate capability to address avian influenza, considering the virus exhibits limited human-to-human transmissibility?

# Author comment: Author response to Decision Letter 1

**DOI:** 10.1371/journal.pgph.0005463.r002

4 Nov 2025

Attachment Submitted filename: rebuttal_letter.docx

# Peer review: Decision Letter 1

**DOI:** 10.1371/journal.pgph.0005463.r003

**Authors:** Sukanta Chowdury

30 Nov 2025

***Quantifying H5N1 outbreak potential and control effectiveness in high-risk agricultural populations***

PGPH-D-25-02162R1

Dear Izel Avkan,

We are pleased to inform you that your manuscript '***Quantifying H5N1 outbreak potential and control effectiveness in high-risk agricultural populations***' has been provisionally accepted for publication in PLOS Global Public Health.

Before your manuscript can be formally accepted you will need to complete some formatting changes, which you will receive in a follow up email. A member of our team will be in touch with a set of requests.

Please note that your manuscript will not be scheduled for publication until you have made the required changes, so a swift response is appreciated.

IMPORTANT: The editorial review process is now complete. PLOS will only permit corrections to spelling, formatting or significant scientific errors from this point onwards. Requests for major changes, or any which affect the scientific understanding of your work, will cause delays to the publication date of your manuscript.

If your institution or institutions have a press office, please notify them about your upcoming paper to help maximize its impact. If they'll be preparing press materials, please inform our press team as soon as possible -- no later than 48 hours after receiving the formal acceptance. Your manuscript will remain under strict press embargo until 2 pm Eastern Time on the date of publication. For more information, please contact globalpubhealth@plos.org.

Thank you again for supporting Open Access publishing; we are looking forward to publishing your work in PLOS Global Public Health.

Best regards,

Sukanta Chowdury, Ph.D

Academic Editor

PLOS Global Public Health

**********
